# Editorial: Secondary antibody deficiency

**DOI:** 10.3389/fimmu.2026.1993941

**Published:** 2026-09-23

**Authors:** Patrick FK Yong, Alexandros Grammatikos

**Affiliations:** 1 Department of Immunology, Frimley Health NHS Foundation Trust, Frimley, United Kingdom; 2 Department of Immunology, North Bristol NHS Trust, Bristol, United Kingdom

**Keywords:** immunodeficiency, antibody deficiency, guidelines, immune deficiency, immunosuppressants, infection, secondary antibody deficiency

Is research into, and recognition of, the impact of secondary antibody deficiency and secondary immune deficiency now reaching a coming of age? That is a question to consider when reading the articles within this important Research Topic in Frontiers. Secondary antibody deficiencies are an acquired condition where there is a quantitative or qualitative reduction in antibodies due to external factors such as other illnesses, particularly haematological malignancy, immunosuppressive or chemotherapeutics agents, and protein loss.

To date, research and attention in the field of immune deficiency has primarily focussed on inborn errors of immunity, driven by their distinct genetic nature which acts as nature’s knockout model for understanding human immunology. In contrast, secondary antibody deficiencies often have multi-factorial causes and can be much more variable. For example, in a patient with cancer, the antibody deficiency could have multiple contributing factors, including the underlying malignancy, chemotherapy or immunotherapy, radiotherapy, and poor nutritional status. Additionally, there is no well-developed, unified classification system for them, with such patients often being seen by multiple disparate specialties.

Nevertheless, secondary antibody deficiencies are of a magnitude greater in frequency compared to inborn errors of immunity, and contribute to significantly larger morbidity and mortality in the population. Their frequency has been increasing rapidly in recent years, with the more widespread use of biologics, immunotherapeutic agents, chemotherapies, and other treatment options for cancers and autoimmune diseases. Because of these advances in therapy, patients are now living longer with disease in remission or under control, but often with a significant compromise to their immune system. Treatments such as CAR-T cell therapy and bispecifics are becoming more common in the management of haematological malignancy, but often carry a profound and long-lasting immunosuppressive effect. The array of immunosuppressive therapies is also increasing rapidly in autoimmune and neurological disease. Further experimental therapies such as trispecifics and BTK degraders are in the pipeline as well.

The above factors make raising awareness and better understanding of how to diagnose and treat secondary antibody deficiency even more pertinent. First, there is the issue of recognition and diagnosis of secondary antibody deficiency. Despite the widespread knowledge that certain medications and health conditions can cause secondary antibody deficiency, it is unfortunately not infrequent that appropriate screening is not undertaken. In their article, Elkhalifa et al. discuss the need for greater awareness of the importance of monitoring for secondary antibody deficiency in patients susceptible to it, and propose ways to identify them. In the same direction, Lagnese et al. propose a diagnostic algorithm that may be used regardless of the underlying aetiology in these patients.^2^ They also discuss the need for further research into predicting the likelihood of secondary antibody deficiency, as well as into which of patients will become susceptible to infection.

Even when secondary antibody deficiency is recognised, management is not always appropriate, with both unnecessarily delayed and over-zealous treatment occurring in practice. There are various contributory factors to this, including the variable training received and experience in secondary antibody deficiency among different specialties that will see these patients, as well as the lack of universally accepted guidelines on how to manage them. In this manner, Elkhalifa et al. summarise potential treatment strategies for these patients, as recommended by the various national and international guidelines. Lagnese et al. offer another perspective into the management algorithms for these patients. Immunoglobulin therapy, which is a key treatment for secondary antibody deficiency, remains in limited supply and hence it is vital to determine which patients will benefit the most from it.

Some more nuanced points within this Research Topic are that there can be an overlap between primary and secondary immune deficiency, as exemplified in the case report by Johnston et al. In this, a patient who presented with recurrent lower respiratory tract infections and borderline hypogammaglobulinaemia is described. The patient was initially diagnosed with anticonvulsant-induced secondary antibody deficiency, but on further evaluation the diagnosis was changed to RNU4ATAC-associated Roifman syndrome. This case highlights the importance of keeping an open mind when evaluating patients with immunodeficiency, and of not hesitating to review the diagnosis in them.

Finally, the article by Luo et al. focuses on the infectious complications that can be seen in patients with secondary antibody deficiency. They present a case of hepatitis B virus reactivation following rituximab therapy in a patient receiving maintenance hemodialysis. This case highlights the importance of keeping a high index of suspicion for infections in these patients, and of the importance of using appropriate screening methods.

In conclusion, it is important to emphasize that secondary antibody deficiency needs to be viewed as a predictable and manageable complication of the modern therapies that we have, rather than as a delayed afterthought. Although secondary antibody deficiency is gaining greater prominence, there is still much work to be done to improve the care of patients affected by it. It is also important that we improve training and develop universally accepted management protocols for these patients. As the number of therapies that suppress the immune system continues to rise, it is critical that their use begins to incorporate protection of the immune function as part of their standard delivery package.

